# Live Attenuated Influenza Vaccination Before 3 Years of Age and Subsequent Development of Asthma: A 14-year Follow-up Study

**DOI:** 10.1097/INF.0000000000001783

**Published:** 2017-09-12

**Authors:** Roger P. Baxter, Ned Lewis, Bruce Fireman, John Hansen, Nicola P. Klein, Justin R. Ortiz

**Affiliations:** From the * Kaiser Permanente Vaccine Study Center, Oakland; † Initiative for Vaccine Research, World Health Organization, Geneva, Switzerland.

**Keywords:** influenza vaccines, vaccine safety, live-attenuated influenza vaccines, asthma

## Abstract

**Background:**

Live-attenuated influenza vaccines (LAIVs) are not licensed in children younger than 2 years of age because of a wheezing safety signal that has not been fully elucidated. In 2000, the Kaiser Permanente Vaccine Study Center conducted a placebo-controlled randomized clinical trial (RCT) of LAIV in children. As many of these children were still enrolled in Kaiser Permanente in 2014, we could assess the possible long-term association between LAIV and subsequent asthma diagnosis.

**Methods:**

We identified all children who were originally enrolled into the LAIV RCT at younger than 3 years of age. We followed up subjects until disenrollment from the health plan, a first diagnosis of asthma, or through the end of the study period in 2014. Asthma was defined by a first International Classification of Diseases, 9th revision, Clinical Modification code (493.*) assigned at an outpatient or emergency department encounter. We performed a survival analysis of time to first asthma diagnosis among children receiving LAIV or placebo with a Cox proportional hazards model.

**Results:**

We identified 1151 children in the original RCT who were 12 through 35 months of age at the time of enrollment and who had received 2 doses of LAIV or placebo. A total of 767 (66.7%) RCT participants were still Kaiser Permanente Northern California members in 2014. There was no evidence of differential dropout by treatment group. The hazard ratio for new-onset asthma for LAIV recipients compared with placebo was 1.1 (95% confidence interval: 0.88–1.41; *P* = 0.38).

**Conclusions:**

We found no evidence of increased risk of subsequent asthma diagnosis among children younger than 3 years of age who received LAIV compared with placebo.

Live-attenuated influenza vaccines (LAIVs) can be an important intervention to prevent severe influenza illness globally. LAIVs have several potential advantages over injectable influenza vaccines. Favorable production speed and yields, ease of administration and demonstrated superiority to inactivated influenza vaccines in children in head-to-head randomized clinical trials (RCTs) indicate that LAIVs should be considered for use in routine national immunization programs.^1,2^ For these reasons, the World Health Organization (WHO) has supported transfer of LAIV manufacturing technology to increase developing country vaccine production capacity for use against seasonal and pandemic influenza.^1^

LAIVs are not currently licensed for use in children younger than 2 years of age,^3^ given concerns of a possible association between LAIV and wheezing in this age group.^2,4,5^ The lack of available LAIVs for children younger than 2 years of age is a major limitation to disease prevention in low- and middle-income countries (LMICs), given that this age group has the greatest pediatric influenza disease burden and the strongest vaccine delivery infrastructure and systems.^3^

There are currently 2 different LAIV technologies in use: (1) Ann Arbor backbone-LAIV produced by AstraZeneca in the United Kingdom, licensed under the names FluMist (United States/Canada) and Fluenz (European Union/European Economic Area), which is licensed in North America and Europe, respectively; and (2) Russian backbone-LAIV produced in Russia (Ultravac) and India (Nasovac-S) and used primarily in those 2 countries. The wheezing signal is specific to the Ann Arbor vaccines.

In 2014, WHO convened an expert consultation to assess LAIV’s potential to prevent pediatric influenza disease in LMICs.^3^ The consultation identified prevention of severe influenza illness in children younger than 2 years of age as an unmet global health need. Participants noted that the mechanism for the safety signal was unknown and needed further elucidation but could be related to reports of early-life respiratory virus infection causing subsequent asthma illness in children.^6^ If the safety signal is real, the risk of LAIV receipt in children younger than 2 years of age may not outweigh the potential benefits. As the expert consultation recommended careful age de-escalation of LAIVs into <2-year age groups as a potential strategy to address the unmet global health need, more date are needed regarding the long-term respiratory health of young children vaccinated with LAIVs. To inform decisions regarding LAIV age de-escalation trials, we conducted this study to assess whether early childhood LAIV vaccination was associated with long-term asthma illness.

## MATERIALS AND METHODS

In 2000, the Kaiser Permanente Vaccine Study Center conducted a placebo-controlled RCT of Ann Arbor LAIV in children.^5^ Children received 2 doses of study vaccine in a 2:1 ratio with placebo. A history of asthma was an exclusion criterion for this trial. Post hoc analyses showed elevated risk ratios in some comparisons of LAIV receipt and asthma, all in children 18–35 months of age.

For our current analysis, we identified all children who were originally enrolled into the LAIV RCT at younger than3 years of age. Three children were not included because they were “live-in” members of another Kaiser Region but living in Northern California at the time of the RCT. Tracking membership over time for those 3 children is problematic, because we may not have had a precise date if they returned to their “home region.” Our exposure of interest was receipt of LAIV or placebo. We followed up subjects from enrollment through 2014 for asthma outcomes. We defined asthma by a first International Classification of Diseases, 9th revision, Clinical Modification code (493.*) assigned at an outpatient or emergency department encounter in the electronic medical record. We performed a survival analysis of time to first asthma diagnosis with a Cox proportional hazards model. We estimated product-limit survival functions with corresponding 95% Hall–Wellner confidence bands. Analyses used SAS software version 9.2 (SAS Institute, Inc, Cary, NC). The study was reviewed and approved by the Kaiser Permanente Northern California (KPNC) Institutional Review Board and the WHO Ethical Review Committee.

## RESULTS

We identified 1151 children in the original RCT who were 12 through 35 months of age at the time of enrollment, who had received 2 doses of the same treatment (LAIV or placebo) and who were enrolled in KPNC at the time of the trial. These 1151 subjects were followed up until they dropped from membership or until they received a first asthma diagnosis. Of included subjects, 503 (43.6%) were 12–23 months and 651 (56.4%) were 24–35 months old. A total of 762 (66.2%) subjects received LAIV, and 389 (33.8%) subjects received placebo. Subjects included 564 (51.0%) males and 587 (49.0%) females. Two thirds (767/1151) of the study population were still KPNC members in 2014. There was no evidence of differential drop-out by treatment group. In any given year, there was never >0.8% deviation from the 2:1 randomization ratio.

Screening for a history of asthma in the original RCT was done by in-person parent interview. Despite this, some subjects had a history of an asthma diagnosis code identified after the trial. However, the proportion was equal between the LAIV and placebo groups, with 70 (9.2%) LAIV recipients and 35 (9.0%) placebo recipients having an asthma diagnosis at any time before study entry.

**FIGURE 1.**
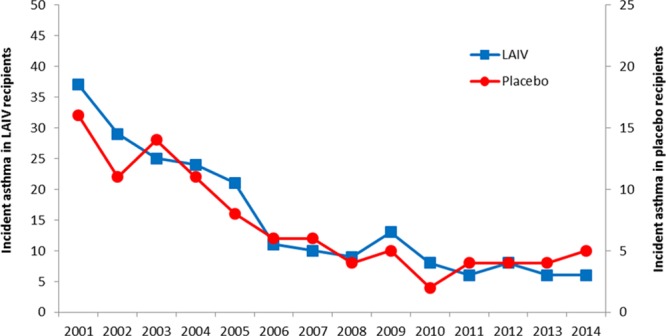
Incident asthma cases in continuously enrolled subjects by LAIV and placebo group, Kaiser Permanente Northern California, 2001–2014. Axes have been adjusted to reflect the 2:1 enrollment ratio.

**FIGURE 2.**
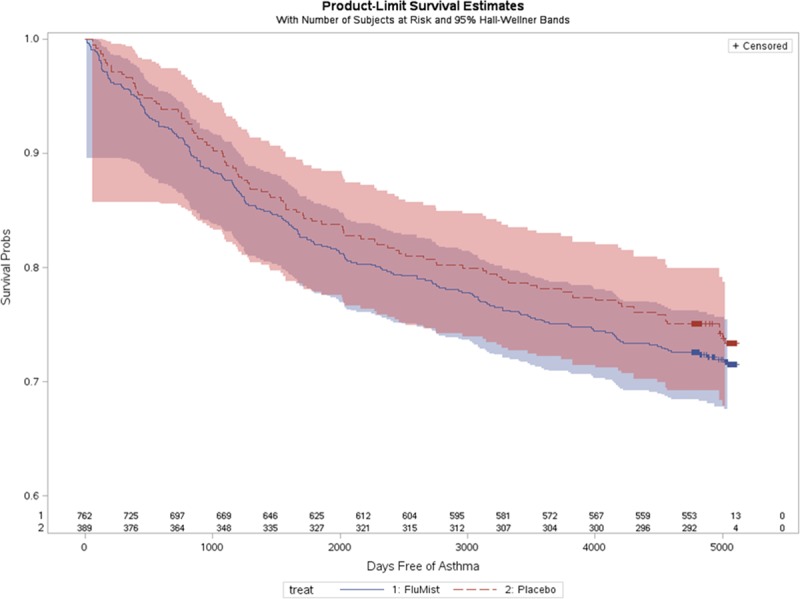
Estimated survival curves of time to first diagnosis of asthma by LAIV and placebo groups, Kaiser Permanente Northern California, 2001–2014. Asthma-free survival among LAIV recipients is shown by the dark blue line, with surrounding 95% CI in light blue. Asthma-free survival among placebo recipients is shown by the red line, with surrounding 95% CI in light red. The substantial overlap of 95% CIs is purple in color.

We graphed the total numbers of LAIV and placebo recipients who had a new (incident) asthma diagnosis by year, adjusting for the 2:1 enrollment ratio (Fig. 1). There were similar asthma diagnosis rates between study groups. A log-rank homogeneity test did not find evidence for nonproportional hazards over time (*P* = 0.41). A proportional hazards model was fit, including only sex and treatment group as independent variables. The hazard ratio for new-onset asthma for LAIV recipients compared with placebo recipients was 1.1 [95% confidence interval (CI): 0.88–1.41; *P* = 0.38]. Female sex was modestly protective, hazard ratio is 0.8 (95% CI: 0.64–0.99; *P* =0.044). Figure 2 shows an estimated survival curve with time to first diagnosis of asthma by study group. The 95% CIs overlapped substantially. We found no evidence of increased risk of subsequent asthma diagnosis among children who received LAIV compared with placebo after 14-year follow-up.

## DISCUSSION

Our study demonstrates that children receiving a dose of LAIV before 3 years of age are no more likely to receive an asthma diagnosis in the subsequent 14 years than children receiving placebo. Previously, researchers have noted that acute wheezing during early childhood respiratory virus infections is a risk factor for subsequent asthma diagnoses.^6^ Despite the studies identifying a safety signal linking LAIV and acute wheezing among children receiving Ann Arbor LAIV,^4,5,7^ it is reassuring that we did not find evidence of association between LAIV exposure and subsequent asthma diagnoses.

The 2000 Kaiser Permanente Vaccine Study Center study was a randomized, double-blind, placebo-controlled clinical trial of LAIV in healthy children 12 months to 17 years of age.^5^ A total of 9689 children were enrolled in the study from October 2000 through December 2000. LAIV was found to be safe in all prespecified safety analyses. Overall, asthma diagnoses in this trial were observed in 0.9% of LAIV recipients and 0.9% of controls. However, elevated risk ratios were observed in 4 of 31 separate post hoc comparisons for asthma among children 18–35 months of age. A subsequent open-label, nonrandomized trial of LAIV among healthy children 18 months to 18 years of age found a significantly increased risk of asthma (relative risk is 2.85; 95% CI: 1.01–8.03) 15–42 days after LAIV among children 18 months to 4 years of age only in the first year of the study.^7^ However, an RCT comparing the safety of LAIV with inactivated influenza vaccine among children 6–59 months of age found that among previously unvaccinated children, wheezing within 42 days was more common after the first dose of LAIV than that of inactivated influenza vaccine,^4^ and this was most pronounced in children younger than 12 months of age. Wheezing illness after vaccine receipt was generally mild and self-limited in all of the above studies. Notably, the Russian LAIV has not been associated with an increase in wheezing illness. Early clinical studies in young children did not include wheezing as a solicited adverse event,^8^ but recent RCTs have not identified a similar signal.^9–11^

Our study provided the unique opportunity to follow up children randomized to receive LAIV or placebo for 14 years in an electronic patient record for asthma diagnoses. The study is not without its limitations, however. Our analysis did not assess long-term safety outcomes aside from asthma. We relied on International Classification of Diseases, 9th revision, diagnostic codes for asthma, which has variable accuracy in children (sensitivity: 44%–92%; specificity: 80%–94%) depending on disease severity, remission and relapse and the reference standard.^12^ However, incorrect asthma diagnosis would have resulted in nondifferential misclassification, given the randomization in the original RCT. Our analyses did not account for subsequent LAIV or other influenza vaccine receipt. However, as LAIV was not licensed for use until 3 years after this study,^13^ none of the study participants in either study group had a second exposure to LAIV until 4 years of age at the earliest, an age thought to be outside the risk window for potential respiratory virus–caused asthma illness.^6^ While we were unable to detect any association between LAIV use and asthma, we could not prove that no such association exists.

It has been estimated that >99% of global pediatric influenza deaths occur in LMICs,^14^ settings in which influenza vaccines are not routinely used.^15^ Given this unmet public health need, WHO has developed Preferred Product Characteristics for Next-Generation Influenza Vaccines.^16^ LAIVs may have characteristics that would make them suitable for use in LMICs to prevent severe influenza disease, but more work needs to be done. Recent observational research from the United States indicate decreased relative effectiveness of Ann Arbor LAIV compared with injectable influenza vaccines in children.^17^ However, the United Kingdom and Finland report that Ann Arbor LAIV performance in the same years and age groups met program expectations.^17^ Similarly, Russian LAIV has also experienced mixed results, with clinical efficacy demonstrated in an RCT among children in Bangladesh but not in a similar study in Senegal.^11^ Better understanding about issues affecting LAIV performance is needed, and corrections, if necessary, must be made before advancing the product into LMICs. While wheezing signals have been found with Ann Arbor LAIV in the past, the benefit–risk calculation may be different with this vaccine in high disease burden settings in which severe illness prevention could outweigh the risk of mild wheezing.^16^ This study supports the 2014 WHO consultation, which recommended that careful age de-escalation studies of children younger than 2 years of age to assess the benefit–risk of LAIVs in LMICs are unlikely to put the children at increased risk of chronic respiratory disease.^3^

## ACKNOWLEDGMENTS

The authors thank Niranjan Bhat, Mark Katz and Margaret Rennels, who provided peer-review of the study protocol for the WHO. The authors would like to acknowledge the contributions of the Centers for Disease Control and Prevention, which provides financial support to the World Health Organization Initiative for Vaccine Research (U50 CK000431).
